# Irrelevant task suppresses the N170 of automatic attention allocation to fearful faces

**DOI:** 10.1038/s41598-021-91237-9

**Published:** 2021-06-03

**Authors:** Haoran Dou, Limei Liang, Jie Ma, Jiachen Lu, Wenhai Zhang, Yang Li

**Affiliations:** 1 Institute for Brain and Psychological Sciences, Sichuan Normal University, Chengdu, 610068 China; 2 College of Education Science, Hengyang Normal University, Hengyang, 421002 China; 3 School of Psychology, Chengdu Medical College, Chengdu, 610500 China; 4 Research Center of Brain and Cognitive Neuroscience, Liaoning Normal University, Dalian, 116029 China; 5 Faculty of Education and Psychology, University of Jyvaskyla, Jyvaskyla, 40014 Finland; 6 School of Psychology, South China Normal University, Guangzhou, 510631 Guangdong China

**Keywords:** Attention, Emotion

## Abstract

Recent researches have provided evidence that stimulus-driven attentional bias for threats can be modulated by top-down goals. However, it is highlight essential to indicate whether and to what extent the top-down goals can affect the early stage of attention processing and its early neural mechanism. In this study, we collected electroencephalographic data from 28 healthy volunteers with a modified spatial cueing task. The results revealed that in the irrelevant task, there was no significant difference between the reaction time (RT) of the fearful and neutral faces. In the relevant task, we found that RT of fearful faces was faster than that of neutral faces in the valid cue condition, whereas the RT of fearful faces was slower than that of neutral faces in the invalid cue condition. The N170 component in our study showed a similar result compared with RT. Specifically, we noted that in the relevant task, fearful faces in the cue position of the target evoked a larger N170 amplitude than neutral faces, whereas this effect was suppressed in the irrelevant task. These results suggest that the irrelevant task may inhibit the early attention allocation to the fearful faces. Furthermore, the top-down goals can modulate the early attentional bias for threatening facial expressions.

## Introduction

The fearful facial expression carries wealthy social information and biological values, indicating a potential threat that requires individuals’ immediate attention^1^. In the natural environment, individuals are more sensitive to others’ fearful expressions to avoid or eliminate the threats. Recently, a number of scholars have concentrated on the attentional bias to fearful faces compared with neutral faces in some attention-related tasks^2,3^. Studies on the basis of functional magnetic resonance imaging (fMRI) have used emotional stimuli and non-emotional stimuli. These studies reported that the amygdala is implicated in the detection of non-emotional features, such as stimulus saliency, stimulus intensity, or experiential arousal^4–7^. For instance, Bishop et al.^6^ found that low-anxious participants showed a reduced amygdala response to unattended versus attended fearful faces, while high-anxious participants exhibited no such reduction, with an increased amygdala response to fearful faces versus neutral faces regardless of attentional focus. Top-down processing—where prefrontal cortex plays a key role—may also modulate the processing of emotional-visual attention^8–11^. The debate has not yet reached a consensus on whether top-down goals could modulate the attentional bias from bottom-up processing of fearful faces and the early neural processing requires further experimental research.

A growing amount of the psychological evidence supported that prior attention to threat stimuli is modulated by top-down signals^3,12–14^. Specifically, the contingent capture hypothesis suggests that contingent attentional capture occurs when a stimulus property captures an observer’s attention, mainly related to the observer’s top-down attentional set for target-defining properties^15^. For instance, using a visual search paradigm, Hahn and Gronlund^12^ demonstrated that a threatening facial expression may guide attention as a high-priority stimulus in the absence of a specific goal; however, in the presence of a specific goal, the efficiency of facial expression search is dependent on the combined influence of a top-down goal and the stimulus characteristics. However, there is no consensus about the top-down modulation of attentional bias for threatening facial expressions. Additionally, Bacon and Egeth^16^ suggested that there are two distinct selection modes, including the singleton detection mode and the feature search mode. They found that goal-directed selection in the feature search mode may override the stimulus-driven capture of salient singletons. We, in the current study, aimed to investigate how different top-down goals may affect the attentional bias to threat faces under a specific search mode.

Several paradigms have been employed to explore the attentional bias for threatening facial expressions, such as the visual search task^12,17,18^, the emotional Stroop task^19,20^, the dot-probe paradigm^21,22^, and the spatial cueing paradigm^14,15,23,24^. In the visual search task and the emotional Stroop task, the stimuli are presented simultaneously, so that the stimulus-driven and goal-driven attention cannot be easily separated^25^. In the dot-probe paradigm, threat-related and neutral stimuli typically appear at the same time. Although participants understand their current goal-driven task, stimuli-driven distractors are always present; thus, the strength of top-down processing is different to control, and the results using this paradigm have a certain one-sidedness^26^. In contrast, in the Posner cueing paradigm^15^, there is a cue after the fixation, and participants then respond to the target. The target location is either consistent with the cueing location (valid cueing) or inconsistent (invalid cueing)^27^. Generally, in the valid cue condition, the target is found faster than that in the invalid cue condition^28^, and the strength of top-down controls in the valid cue condition is weaker than that in the invalid cue condition^29^. Moreover, other studies highlighted the association between the current task and threat-related stimuli to control top-down processes. According to the findings of previous studies, when a task is relevant to the emotion, threat-related stimuli have a special propensity to attract; when the task is irrelevant to the emotion, the attentional bias toward threat-related stimuli is inhibited^3,14^. Victeur et al.^30^ pointed out that attentional allocation to irrelevant fearful faces is conditional to the explicit relevance of fearful expressions to top-down search goals.

Event-related Potentials (ERPs) are high resolution indices of automatic and conscious processing. The N170 ERP component is a right hemisphere lateralized negativity peaking around 170 ms after stimulus-onset, as well as being a reliable marker of face detection^31,32^. A number of scholars pointed out that facial expressions are also related to N170 during early processing, indicating that emotional faces can induce larger N170 amplitudes than neutral faces^33–35^. Moreover, previous studies have reported that the N170 represents an early stage of visual processing of face that can be modulated by an interaction between the task and the diagnosticity of the stimulus for the task^31,36–38^. In contrast, the vertex positive potential (VPP) is a positive peak recorded in fronto-central sites, and can reflect the same neural processes as N170^39^. A previous research demonstrated that similar to N170, facial expression modulates VPP amplitude and its amplitude is larger in response to fearful faces relative to happy and neutral faces^40^. However, other studies indicated that VPP may be attenuated in some conditions, such as faces disrupted by an inversion or scramble^41,42^, which is different from N170. Joyce and Rossion^43^ argued that controversial results of N170 and VPP reported in previous studies could be attributable to the location of the reference electrode.

In the current study, we aimed to use N170 and VPP as electrophysiological indicators to examine whether and to what extent top-down goals can influence attentional bias for threatening facial expressions. Previously reported behavioral and fMRI findings indicated that the cue validity and task relevance can modulate the top-down control of attention with the similar activations in the parietal lobe^44–51^ and the definition contains both perception set and task set^52^. Therefore, in the present study, to effectively detect the extent of top-down control interplaying with the emotional bottom-up processing, we combined the two factors influencing top-down control. More specifically, we used a modified spatial cueing task according to cue validity (invalid or valid cue) and task relevance (emotion or gender recognition). Based on previous results^3,14,53,54^, we hypothesized that the attentional bias for fearful faces may appear in the relevant task and may disappear in the irrelevant task. Similarly, there might be significant differences between fearful faces and neutral faces on the amplitudes of N170 and VPP in the relevant task. However, such differences may be inhibited due to the difficulty in disengagement in the irrelevant task.

## Results

### Reaction time (RT)

**Figure 1:**
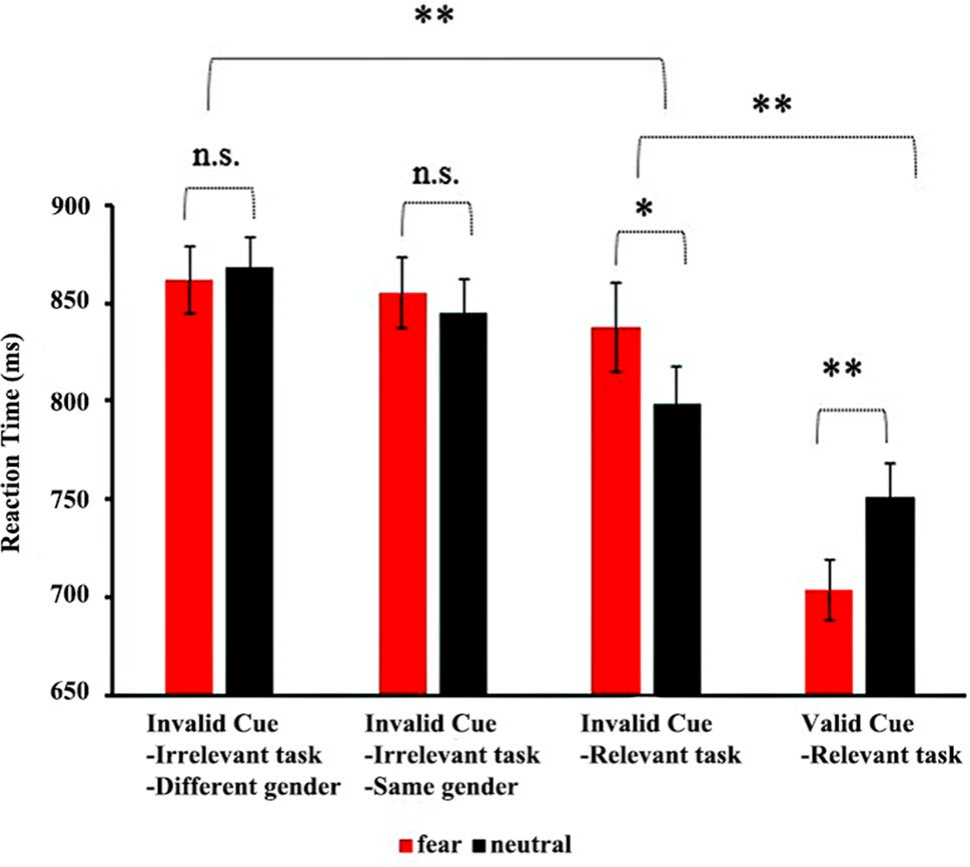
Reaction time for different top-down goals with fearful and neutral faces; *P < 0.05, **P < 0.01.

The outliers of RT outside the range of ± 3 *SDs* from the mean were excluded from our analysis. The two-way repeated-measures ANOVA showed a significant effect of the top-down controls (*F*(3, 81) = 8.00, *P* < 0.01, *η*_*p*_^2^ = 0.54). Post-hoc analyses indicated that participants responded faster to the valid cue-relevant task condition than to the other three conditions (*P* < 0.01). They also responded faster to the invalid cue-relevant task condition than to the invalid cue-relevant task concerning different gender-based conditions (*P* < 0.01). No main effect of cue emotion was detected (*F*(1, 27) = 0.01, *P* = 0.91, *η*_*p*_^2^ = 0.03). In addition, the analysis showed a significant interaction between top-down controls and cue emotion (*F*(3, 81) = 4.91, *P* < 0.01, *η*_*p*_^2^ = 0.36 (see Fig. 1). Simple-effect analyses revealed that the mean RT to the fearful faces (703.31 ± 78.96 ms) was shorter than that to the neutral faces (750.67 ± 63.82 ms) in the valid cue-relevant task condition (*P* < 0.01); in the invalid cue-relevant task condition, the mean RT to the fearful faces (837.72 ± 74.92 ms) was longer than that to the neutral faces (797.99 ± 57.29 ms) (*P* < 0.05). However, in the irrelevant task, no significant difference between the fearful and neutral faces was found in the mean RT.

### Accuracy

We used two-way repeated-measures ANOVA and found that the main effect of top-down controls was significant (F(3, 81) = 17.049, *P* < 0.001, *η*_*p*_^2^ = 0.387). Besides, the pairwise comparison showed that the accuracy of valid cue-relevant task condition was higher than that of other conditions (invalid cue-relevant task condition (*P* < 0.01), two conditions in the irrelevant task (*P* < 0.001)). The main effect of emotion-type was not statistically significant (F(1, 27) = 0.001, *P* > 0.05, *η*_*p*_^2^ = 0.001). The interaction effect between emotion-type and top-down controls was not significant as well (F(3, 81) = 0.400, *P* > 0.05, *η*_*p*_^2^ = 0.015).

### N170 amplitude

**Figure 2:**
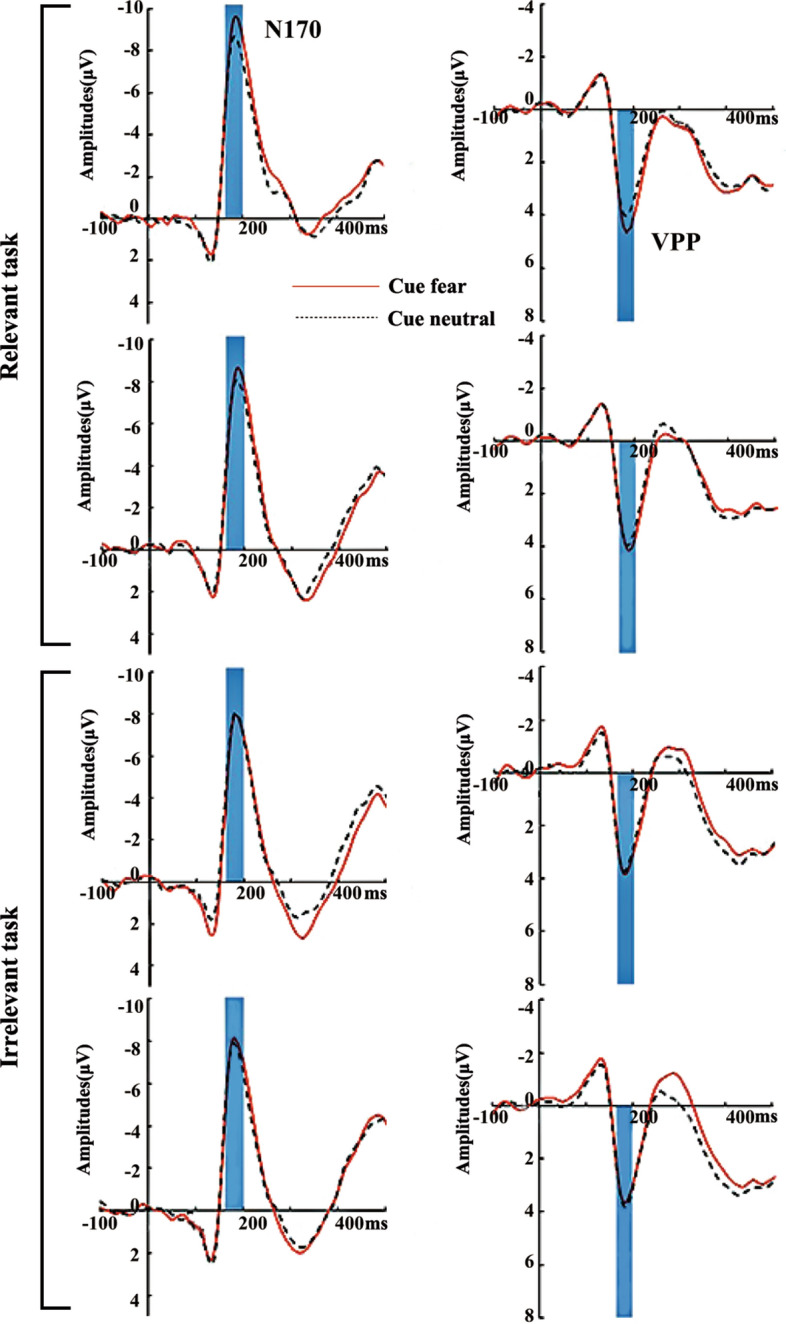
Grand-average waveforms for four top-down controls at ROI of electrodes. (**A**) The fearful faces in the cue position (red solid line) and the neutral faces in the cue position (black dotted line) on the valid cue-relevant task condition. (**B**) The fearful faces in the cue position (red solid line) and the neutral faces in the cue position (black dotted line) on the invalid cue-relevant task condition. (**C**) The fearful faces in the cue position (red solid line) and the neutral faces in the cue position (black dotted line) on the invalid cue-irrelevant task with the same gender-dependent condition. (**D**) The fearful faces in the cue position (red solid line) and the neutral faces in the cue position (black dotted line) on the invalid cue-irrelevant task with different gender-dependent conditions.

**Figure 3:**
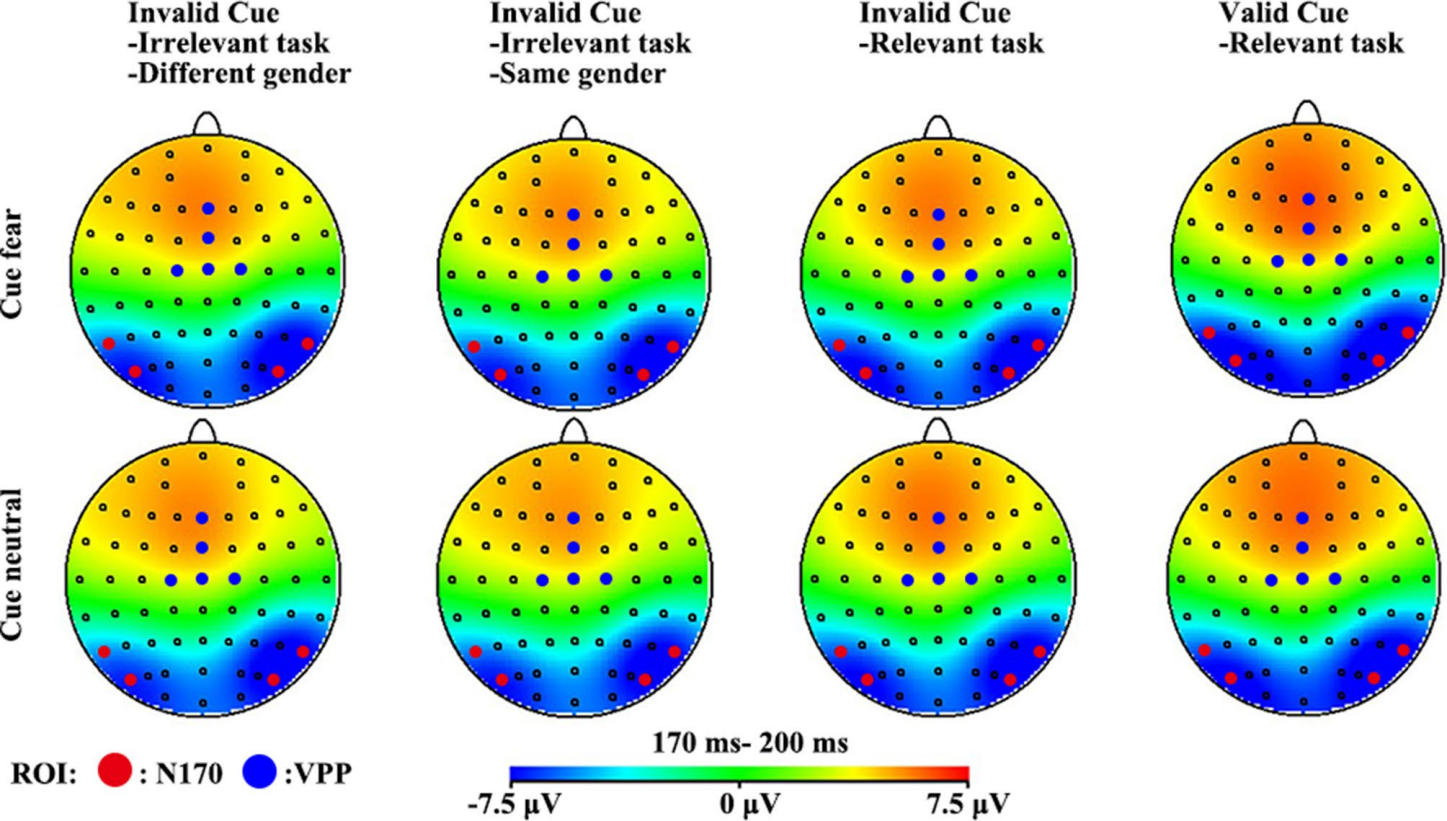
Topographic maps representing the scalp distribution of the N170 and VPP (170–200 ms) for the fearful and neutral faces in four top-down controls, respectively. The figure was plotted by the Brain Vision Analyzer 2 and Photoshop CS6 software.

**Figure 4:**
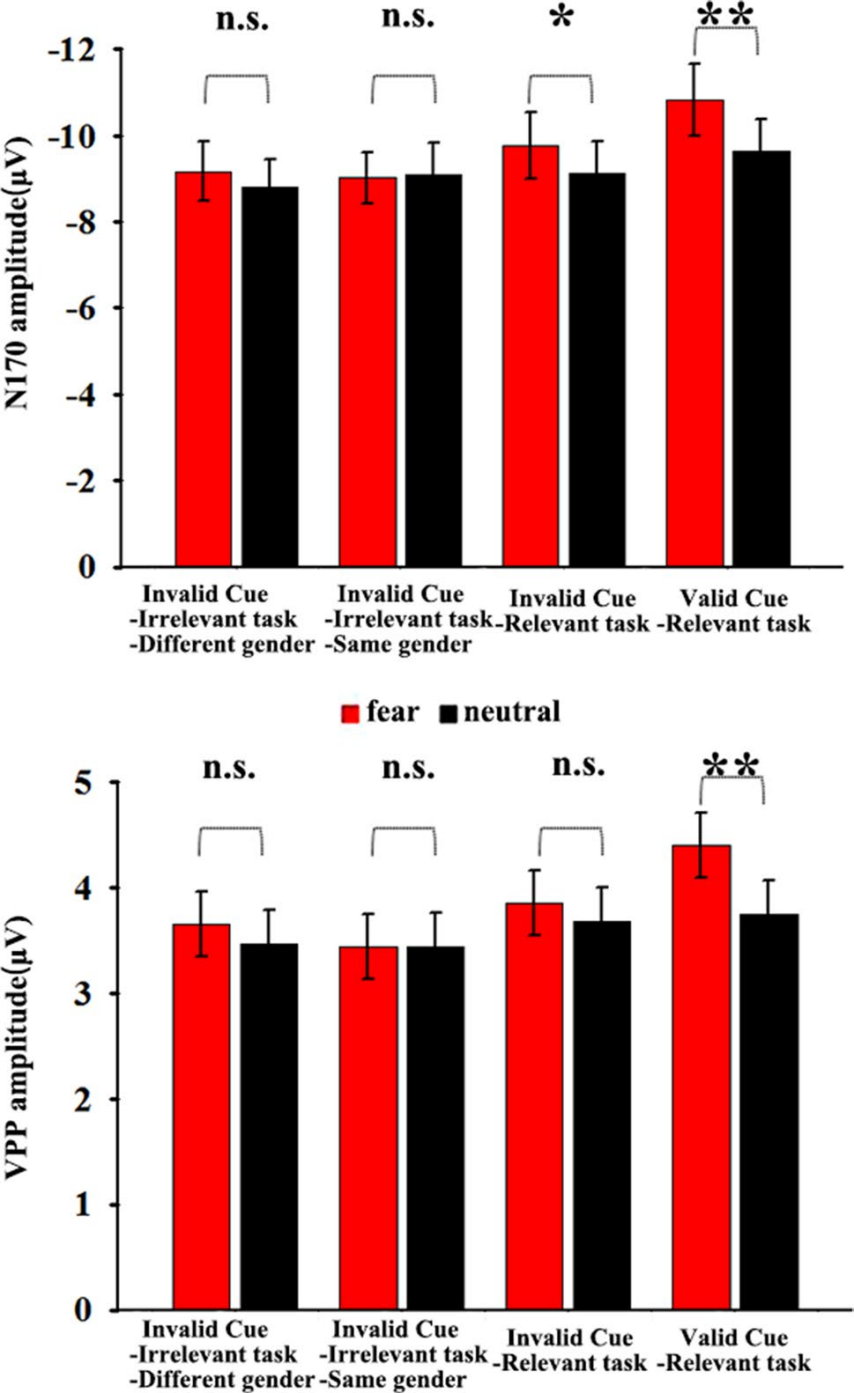
A bar plot reporting the results in the N170 and VPP components in the different top-down controls; *P < 0.05, **P < 0.01.

The analysis performed using N170 amplitude revealed a significant main effect of the emotional cue (*F*(1, 27) = 14.33, *P* < 0.01, *η*_*p*_^2^ = 0.113). The fearful faces evoked a significantly larger N170 compared with the neutral faces (P < 0.01). However, no main effect of top-down controls was identified (*F*(3, 81) = 0.65, *P* = 0.58, *η*_*p*_^2^ = 0.17). Moreover, there was a significant interaction between top-down controls and emotional cue (*F*(3, 81) = 3.67, *P* < 0.05, *η*_*p*_^2^ = 0.09). Simple-effect analyses indicated that N170 amplitude of the fearful faces (− 10.83 ± 0.84 µV) was greater than that of the neutral faces (− 9.63 ± 0.75 µV) in the valid cue-relevant task condition (*P* < 0.01). In addition, N170 amplitude of the fearful faces (− 9.76 ± 0.77 µV) was larger than that of the neutral faces (− 9.13 ± 0.74 µV) in the invalid cue-relevant task condition (*P* < 0.05) (see Figs. 2, 3, 4).

### VPP amplitude

The analysis conducted using VPP amplitude showed a significant main effect of the emotional cue (*F*(1, 27) = 9.76, *P* < 0.01, *η*_*p*_^2^ = 0.078). The fearful faces evoked a larger VPP compared with the neutral faces (*P* < 0.01). However, no main effect of top-down controls was detected (*F*(3, 81) = 0.84, *P* = 0.47, *η*_*p*_^2^ = 0.021). Additionally, a significant interaction between top-down controls and the emotional cue was found (*F*(3, 81) = 2.83, *P* < 0.05, *η*_*p*_^2^ = 0.068). Simple-effect analyses revealed that VPP amplitude of the fearful faces (4.41 ± 0.29 µV) was larger than that of the neutral faces (3.75 ± 0.32 µV) in the valid cue-relevant task condition (*P* < 0.01). However, the VPP amplitude exhibited no significant difference between the fearful faces and the neutral faces in other levels (see Figs. 2, 3, 4).

## Discussion

We applied a modified spatial cueing paradigm to investigate attentional bias to fearful faces under different top-down controls with the irrelevant task and invalid cue. The results showed that fearful faces induced larger N170 amplitudes than neutral faces in the relevant task, however, no significant difference in N170 amplitudes was identified between two faces in the irrelevant task. This result indicated that the difficulty of disengagement from the irrelevant task suppressed the automatic attention allocation of fearful faces. For the behavioral data, in line with previous studies^2,35^, we found that fearful faces attracted more attention than neutral faces in the valid cue-relevant task condition. Moreover, in the invalid cue-relevant task condition, fearful faces exhibited a slower RT compared with neutral faces. Besides, we noted that the attentional bias for fearful faces disappeared in the irrelevant tasks. Taken together, these results suggest that top-down processing could regulate the early attentional bias for fearful faces and modulate the neural processing of facial expressions.

In the current research, we found that top-down processing moderates the automatic attention process of fearful faces. A previous study suggested that emotional faces are prioritized over neutral ones^55^. Folk and Remington^56^ demonstrated irrelevant singletons can produce distraction effects that are dissociable from shifts of spatial attention. This view, known as the contingent capture hypothesis, has been supported by several researches^13,57,58^. According to the contingent capture hypothesis, it can be explained that in the relevant task, fearful faces are defined by special features relevant to the target; thus, fearful faces are able to attract spatial attention. In contrast, fearful faces share few features with target in the irrelevant task, and they can be easily overlooked by a top-down goal. Moreover, we found different emotional effects of RT in the relevant task. In the valid cue-relevant task condition, we noted that the RT of fearful faces was smaller than that of the neutral faces. This result indicated that the fearful faces facilitated the orienting of spatial attention. It was reported that threatening stimuli are prioritized over other stimuli to ensure adaptive behavior, while they do not automatically capture attention, as an attentional effect in both conditions was found^59–61^. However, in the invalid cue-relevant task condition, the RT of fearful faces was higher than that of the neutral faces. This result could be related to the difficulty in disengagement of the fear faces. This controversial outcome was also consistent with the previously reported finding. Carlson and Reinke^62^ demonstrated that fearful eye-whites could be a salient feature of fearful facial expressions that elicit modulations in spatial attention.

For N170 amplitude, a previous research showed that N170 component may reflect the structural encoding of facial faces^63^, and it can be modulated by emotional facial expressions^33,64^. Moreover, Crist et al.^65^ demonstrated that the early face-specific processing is not automatic, and it strongly depends on endogenous factors (e.g., allocation of spatial attention). In the present study, we found significant differences between fearful faces and neutral faces in the weak and the medium conditions, while no differences were identified in strong and very strong conditions. This result was consistent with our hypothesis. The gender-dependent recognition task (irrelevant task) exhibited to have a more complicated top-down goal-directed processing compared with the emotion recognition task. Therefore, the effect of difficulty in disengagement from the fearful faces was suppressed in the irrelevant task, which was consistent with the previously reported results^14,30^. More specifically, Vromen et al.^14^ used the threatening stimuli (spider figure) and spatial cueing paradigm with two different tasks; they also found that the delayed disengagement from a non-target spider was observed only when the spider was the target set, rather than when it was task-irrelevant. Jiang et al.^66^ compared the N170 component between fearful and neutral faces in the Continuous Flash Suppression (CFS) paradigm. Their results revealed that the fearful faces can still evoke larger N170 compared with the neutral faces even in the unawareness condition, while further attention was needed. The results of the current research supported their findings that the attention is the key point in the responses of N170 to the fearful faces.

Besides, our results might also be able to explain by perceptual load theory. According to the perceptual load theory^67^, during the processing of selective attention, the allocation of the attention was decided by perceptual load; the distraction can only be processed in the low perceptual load. In the current study, the irrelevant task (gender-dependent recognition task) had a slightly higher perceptual load than that of the relevant task (emotion recognition task). The N170 showed no significant difference between fearful and neutral faces in the larger perceptual load conditions (in the irrelevant task), while in the lower perceptual load conditions (in the relevant task), the fearful faces evoked larger N170 than the neutral faces. However, it was difficult to compare the difference between the pure perceptual load and the different top-down goal-directed perceptual sets in this study. We therefore suggest the necessity of a new design for the future studies to distinguish the difference between the pure perceptual load and the top-down goal-directed perceptual sets.

We found that VPP, as same as N170, was affected by the different top-down goals. Specifically, fearful faces elicited a larger VPP amplitude than neutral faces in the valid cue-relevant task, and there was no significant difference in other conditions. Moreover, the similar latencies of N170 and VPP indicated the possibility that they are derived from the same neural dipole, which is consistent with findings of previous studies^35,43,68^. Nevertheless, N170 and VPP amplitudes showed differences in the invalid cue-relevant task, which might be related to the fact that the reference electrode might affect the observed signals and functional differences between the N170 and VPP components^43^. For instance, Itier and Taylor^42^ found a larger response of N170 amplitude to inverted faces, whereas this effect was not significant at the level of VPP. In the present study, the average reference was used, which yielded a large peak at N170 sites and a small peak at VPP sites. Therefore, in our study, we could not justify how the VPP and N170 showed distinguished activities in the invalid cue-relevant task condition. Future studies should use further reference sites, e.g. the reference electrode standardization technique, to explore the relationship between the two components and top-down goal-directed effects.

In addition, there were no differences in response time, N170 amplitude, and VPP amplitude between fearful faces and neutral faces in the irrelevant task. These results indicated that the attentional bias for threatening stimuli was suppressed by the irrelevant task. That means the effect of fear facial distraction in the cue position was also inhibited by suppressing the delayed disengagement from fearful faces. However, there was no difference in the accuracy between fearful faces and neutral faces in any conditions. The above-mentioned findings confirmed that the accuracy in this design was not sensitive to reflect the emotional effect compared with other activities (e.g., RT or N170).

There were a number of limitations in the current research. Firstly, we did not counterbalance the facial emotion in the target position between the gender-dependent recognition and emotion recognition tasks. This might associate with some extra variables when two tasks are compared, which can be resolved in the future researches. Secondly, the potential extra variables of a single condition in different tasks might be different in the gender consistency. Thirdly, due to the objectives of the study, it was difficult to compare the “overall” effect from validity or task-irrelevant, and task-irrelevant changes were not considered. Last but not least, the ERPs used in this study were not sensitive to the spatial resolution. Therefore, we did not know the activities in the related brain regions (e.g., fusiform face area). Hence, further studies are warranted to eliminate the above-mentioned deficiencies and confirm our findings.

## Conclusions

In summary, fearful faces facilitated the orienting of spatial attention and difficulty in disengagement from fearful faces in the relevant task, while the disengagement from fearful faces was inhibited in the irrelevant task. Moreover, we provided more early neural processing evidence on the basis of different top-down goals, modulating early stimulus-driven attentional bias during the VPP/N170 time window.

## Materials and methods

### Participants

Twenty-eight healthy college students (14 females; mean age, 22.83 years old, standard deviation (SD) of age, 2.95 years old) from Liaoning Normal University (Dalian, China) participated in the study. They were all right-handed and had normal or corrected-to-normal vision without psychiatric or neurological history. All participants provided written informed consent prior to commencing the study. The study was carried out in accordance with the Declaration of Helsinki^69^ and the research protocol was approved by the Ethics Committee of Liaoning Normal University.

### Stimuli and apparatus

All pictures were selected from the Chinese Facial Affective Picture System (CFAPS)^70^. The pictures included 20 neutral faces and 20 fearful faces, with an equal number of male and female faces. All pictures were frontal headshots. The fearful and the neutral faces differed significantly in valence (mean ± *SD*: fearful = 2.78 ± 0.98, neutral = 4.35 ± 0.12; *t*(38) = 2.98, *P* < 0.01), while those were similar in arousal (mean ± *SD*: fearful = 5.32 ± 0.54, neutral = 5.31 ± 0.27; *t*(38) = 0.72, *P* > 0.05). Each face was displayed within a placeholder box—a black outline with the size of 114 × 88 pixels (i.e., 3 × 2.6 cm^2^). Both spatial cue and target box were green. Therefore, the cue should produce a reliable cueing effect on target identification, even though it was uninformative to the actual target location^14^. The stimuli were presented on a 19-inch monitor with a resolution of 1024 × 768 pixels at a 100 Hz refresh rate. The viewing distance was around 80 cm.

### Task

**Figure 5:**
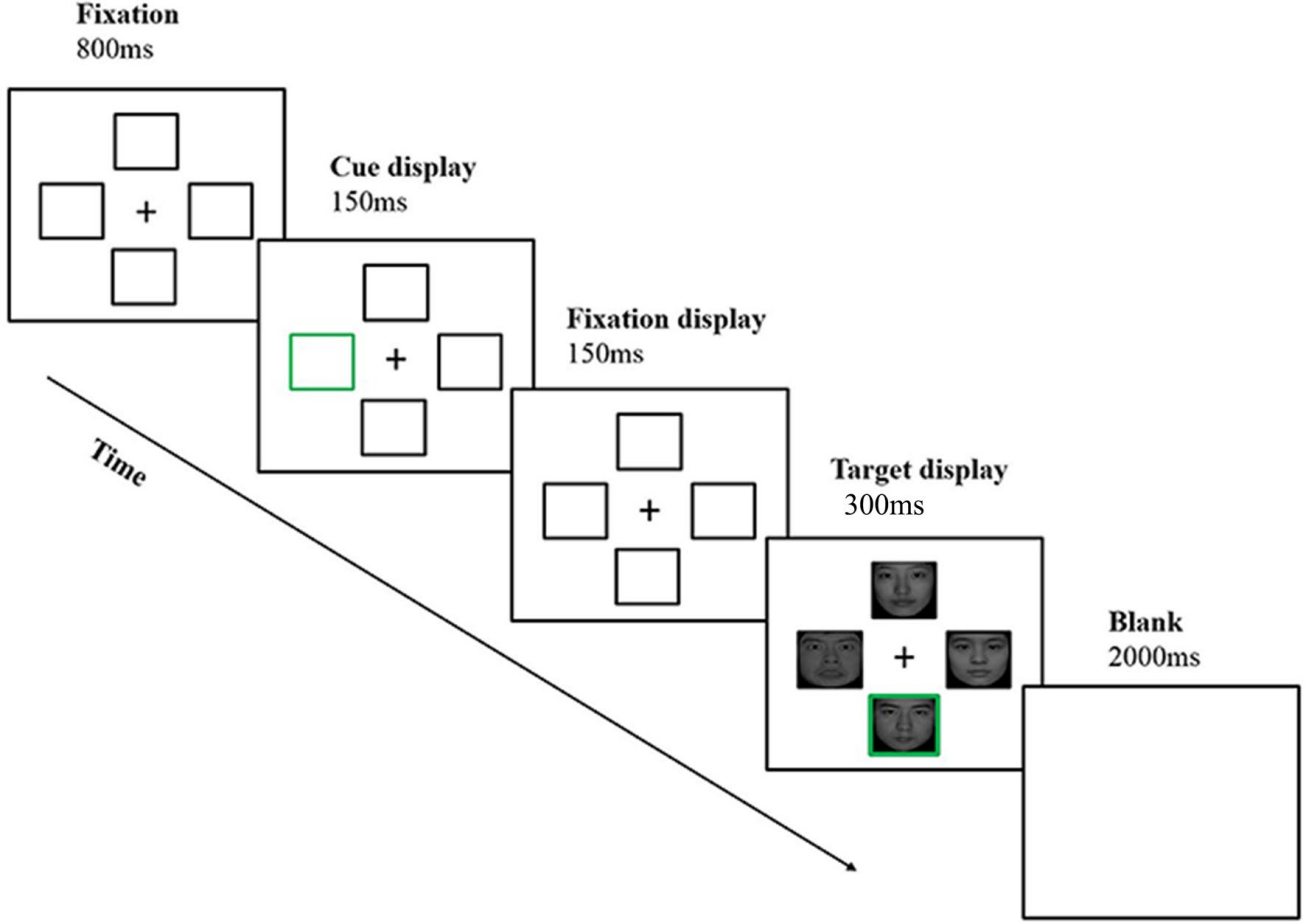
The modified spatial cueing task. A trial in the modified spatial cueing task consisted of the following display sequences: fixation display, cue display, fixation display, target display, and blank screen. The depicted trial represents a fearful face in the strong top-down controls.

We used a modified spatial cueing paradigm and a 4 (top-down controls) × 2 (the emotion-type of the cue position in the target) within-subjects design. Participants were comfortably seated in a quiet laboratory and received instructions to complete the modified spatial cueing task (see Fig. 5). At the beginning of each trial, a central fixation cross and four placeholder boxes were presented for 800 ms in a cross-like arrangement. Each box was positioned with its nearest corner 6 cm away from the central fixation cross. Next, the cue (green frame), one of the four boxes with an equal probability, appeared for 150 ms. When another fixation displayed for 150 ms, the target was presented for 300 ms. The target consisted of the fixation at the center and four facial pictures around. On each trial, there were three non-target faces and one target face with the green frame. Once the target appeared, participants had to respond as quickly and accurately as possible by pressing the “F” or “J” key on a keyboard (emotion recognition task: F, fearful face, J, neutral face; gender-dependent recognition task: F, male, J, female). The keyboard press was counterbalanced between the participants. Each trial ended with the presentation of a blank screen for 2000 ms.

In the emotion recognition task, the participants were asked to distinguish the emotion (fearful or neutral) of the target face. In the valid cue condition, the green frame was displayed at the same position as the cue. In the invalid cue condition, the green frame was not displayed at the cued position. The fearful and neutral faces were equal in terms of cue (target) position.

In the gender-dependent recognition task, the cue and target positions were inconsistent. Moreover, participants were asked to identify the gender (male or female) of the target face rather than recognize its emotion. In the same gender-dependent condition, the cue and target faces were of the same gender. However, in the different gender-dependent conditions, the cue and target faces were of the different genders. In all conditions during the two tasks, there were always two female faces and two male faces, and the distribution of gender within the targets was 50% male and 50% female.

Each participant completed 36 practice trials and 384 test trials. The trials in each of the four different conditions covered 25% (96 trials) of the total number of trials. The four conditions were divided into two blocks according to their task (emotion recognition and gender-dependent recognition). The experimental instructions were presented to participants before each block. In the emotion recognition block, trials of the weak and medium conditions were randomly presented. In the subsequent gender-dependent recognition block, the trials of the strong and very strong conditions were also randomized. There was a period of rest of more than 60 s after every 96 trials.

### Electrophysiological recording and analysis

Electroencephalographic (EEG) data were recorded using 64 Ag/AgCl electrodes placed in a Quick-cap (conforming to the International 10–20 System). The data were referenced online to the CPz electrodes. Horizontal electrooculography (EOG) data were recorded from two electrode sites at the outer canthi of each eye. Vertical EOG data were recorded from electrodes situated on infra-orbital and supra-orbital regions of the left eye. The impedance of the electrodes was kept below 5 kΩ. The sampling rate was 500 Hz. The signal was band-pass filtered at 0–104 Hz and stored for offline analysis. The EEG processing, ERP analysis, and plotting of figures (Figs. 2, 3) were performed using the Brain Vision Analyzer 2 software (Brain Products GmbH, Berlin, Germany). If the electrodes could not collect standard data (e.g., loose or faulty electrodes), we adopted the interpolation method by using the electrodes nearby. In order to remove the artifact of movement, we evaluated raw data manually for obvious drifting and other artifacts before the analysis (< 5% data). A semi-automatic independent-component-analysis-based algorithm was used to perform blink correction^71^. The EEG data were re-referenced off-line to the average reference and band-pass filtered to 0.1–30 Hz (24 dB/octave). The ERP waveforms were time-locked to the onset of the target stimulus, and their time window was from 100 ms pre-stimulus to 1000 ms post-stimulus with a 100 ms pre-stimulus baseline. Epochs with amplitudes over ± 75 µV were automatically rejected from averaging.

In the present study, the peak amplitudes of the N170 and VPP components were analyzed. Based on previously reported findings^72,73^ and the topographical distribution of the grand-average ERP activity, we selected the following electrodes of interest: P7, P8, PO7, and PO8 for N170 in the time window of 170–200 ms; and Fz, FCz, Cz, C1, and C2 for VPP in the time window of 170–200 ms. According to the research conducted by Luck and Gaspelin^65^, we further used the average value of each electrode group to reduce the number of analyses and potential type-I error in the analysis of variance outcomes. Therefore, statistical analysis did not include the electrode-dependent factors.

Two-way repeated-measures analysis of variance (ANOVA) was used to analyze the ERPs and RT as dependent variables and top-down controls and cue emotion at the cue position in the target as within-subjects factors. Degrees of freedom were corrected according to the Greenhouse–Geisser method. In addition, the Bonferroni correction method was utilized for multiple comparisons. All the statistical analyses were conducted using the SPSS 19.0 software (IBM, Armonk, NY, USA).
